# HSP22 (HSPB8) positively regulates PGF2α-induced synthesis of interleukin-6 and vascular endothelial growth factor in osteoblasts

**DOI:** 10.1186/s13018-021-02209-8

**Published:** 2021-01-21

**Authors:** Gen Kuroyanagi, Go Sakai, Takanobu Otsuka, Naohiro Yamamoto, Kazuhiko Fujita, Tetsu Kawabata, Rie Matsushima-Nishiwaki, Osamu Kozawa, Haruhiko Tokuda

**Affiliations:** 1 Department of Orthopedic Surgery, Nagoya City University Graduate School of Medical Sciences, 1 Kawasumi, Micuho-cho, Mizuho-ku, Nagoya, 467-8601 Japan; 2 Department of Rehabilitation Medicine, Nagoya City University Graduate School of Medical Sciences, Nagoya, 467-8601 Japan; 3 Department of Pharmacology, Gifu University Graduate School of Medicine, Gifu, 501-1194 Japan; 4 Department of Orthopedic Surgery, Komaki City Hospital, Komaki, 485-8520 Japan; 5 Department of Clinical Laboratory/Biobank of Medical Genome Center, National Center for Geriatrics and Gerontology, Obu, 474-8511 Japan

**Keywords:** HSP22, PGF2α, IL-6, VEGF, Osteoblast

## Abstract

**Background:**

Heat shock protein 22 (HSP22) belongs to class I of the small HSP family that displays ubiquitous expression in osteoblasts. We previously demonstrated that prostaglandin F2α (PGF2α), a potent bone remodeling factor, induces the synthesis of interleukin-6 (IL-6) and vascular endothelial growth factor (VEGF) via p44/p42 mitogen-activated protein (MAP) kinase and p38 MAP kinase in osteoblast-like MC3T3-E1 cells. In the present study, we investigated whether HSP22 is implicated in the PGF2α-induced synthesis of IL-6 and VEGF and the mechanism of MC3T3-E1 cells.

**Methods:**

MC3T3-E1 cells were transfected with HSP22-siRNA. IL-6 and VEGF release was assessed by ELISA. Phosphorylation of p44/p42 MAP kinase and p38 MAP kinase was detected by Western blotting.

**Results:**

The PGF2α-induced release of IL-6 in HSP22 knockdown cells was significantly suppressed compared with that in the control cells. HSP22 knockdown also reduced the VEGF release by PGF2α. Phosphorylation of p44/p42 MAP kinase and p38 MAP kinase was attenuated by HSP22 downregulation.

**Conclusions:**

Our results strongly suggest that HSP22 acts as a positive regulator in the PGF2α-induced synthesis of IL-6 and VEGF in osteoblasts.

## Introduction

Bone tissue is continuously remodeled to maintain bone homeostasis through bone remodeling, and the bone metabolism is tightly coordinated by two types of functional cells, osteoclasts and osteoblasts [1]. The former cells contribute to bone resorption, whereas the latter cells are responsible for bone formation. The process of bone remodeling is initiated with the osteoclastic resorption of an old bone, and osteoblasts subsequently migrate into the resorption lacuna, leading to the formation of a new bone [2]. Various bone remodeling factors including cytokines and growth factors regulate the bone metabolism [3]. It is well recognized that interleukin-6 (IL-6), a proinflammatory cytokine, is a potent bone resorptive agent, which stimulates osteoclastic bone resorption in bone metabolism [4]. On the other hand, vascular endothelial growth factor (VEGF) stimulates the proliferation of vascular endothelial cells as a specific mitogen [5]. Evidence is accumulating that the microvasculature provided by capillary endothelial cells is essential for bone remodeling [6]. Thus, it is currently recognized that the functions of osteoclasts, osteoblasts, and capillary endothelial cells are strictly coordinated by one another, and the three types of cells cooperatively drive bone metabolism. In our previous studies [7, 8], we have shown that prostaglandin F2α (PGF2α), a potent bone remodeling mediator [9], induces the synthesis of IL-6 and VEGF in osteoblast-like MC3T3-E1 cells. Regarding the intracellular signaling of PGF2α, we demonstrated that p44/p42 mitogen-activated protein (MAP) kinase and p38 MAP kinase are involved in the synthesis of IL-6 and VEGF in these cells [8, 10–12].

Heat shock proteins (HSPs) recognized as molecular chaperones are induced in the cells in response to environmental stresses including heat and oxidation [13]. It is generally established that HSPs act as key regulators of proteostasis under the stress conditions [13]. The HSP family is classified into seven groups, namely HSPA (HSP70), HSPB, HSPC (HSP90), HSPD/E (HSP60/HSP10), HSPH (HSP110), DNAJ (HSP40), and CCT (TRiC) [13, 14]. Among the seven groups, HSPBs are known as small molecular weight HSPs with molecular mass in the range of 12–43 kDa, and ten small HSPs have been identified [13]. Accumulating evidence indicates that the small HSP family is classified into class I (ubiquitous expression) and class II (tissue-restricted expression) [13, 15]. Thus, it is currently recognized that ubiquitously expressed small HSPs are involved in various cellular processes such as vasoconstriction and cancer in addition to protein folding [13].

Heat shock protein 22 (HSP22; HSPB8) that belongs to class I is expressed abundantly in the muscle, heart, and brain [16–18]. With regard to HSP22 in diseases, it has been reported that neuromuscular diseases including distal hereditary motor neuropathy and Charcot-Marie-Tooth disease are caused by the dysfunction of HSP22 [19, 20]. Additionally, HSP22 reportedly regulates the progression of cancers such as glioblastoma, melanoma, and breast cancer [21, 22]. As for HSP22 in osteoblasts, we have previously demonstrated that HSP22 exists in quiescent osteoblast-like MC3T3-E1 cells and plays a limiting role in the cell migration stimulated by transforming growth factor-β [23]. In our recent study, we have shown that downregulation of HSP22 reduces tumor necrosis factor-α-stimulated IL-6 synthesis [24]. However, the exact roles of HSP22 in osteoblasts remain to be clarified.

In this study, we investigated whether HSP22 is involved in the PGF2α-induced synthesis of IL-6 and VEGF in osteoblast-like MC3T3-E1 cells. We herein demonstrate that HSP22 acts as a positive regulator in the synthesis of IL-6 and VEGF through p44/p42 MAP kinase and p38 MAP kinase in these cells.

## Materials and methods

### Materials

PGF2α was obtained from Sigma-Aldrich Co. (St. Louis, MO, USA). The ELISA kits for mouse IL-6 and mouse VEGF were purchased from R&D Systems, Inc. (Minneapolis, MN, USA). Phospho-specific p44/p42 MAP kinase antibodies, p44/p42 MAP kinase antibodies, phospho-specific p38 MAP kinase antibodies, and p38 MAP kinase antibodies were obtained from Cell Signaling Technology, Inc. (Danvers, MA, USA). Glyceraldehyde-3-phosphate dehydrogenase (GAPDH) antibodies were purchased from Santa Cruz Biotechnology, Inc. (Santa Cruz, CA, USA). An ECL Western blotting detection system was obtained from GE Healthcare Life Sciences (Buckinghamshire, UK). Negative control-small interfering RNA (siRNA) (Silencer Negative Control #1 siRNA (Neg)) and HSP22-siRNA (s206904 (#1) and s96094 (#2)) were purchased from Ambion (Austin, TX, USA). Other materials and chemicals were obtained from commercial sources.

### Cell culture

Cloned osteoblast-like MC3T3-E1 cells which had been derived from newborn mouse calvaria [25] were maintained as described previously [7]. In brief, the MC3T3-E1 cells were cultured in α-minimum-essential medium (α-MEM) containing 10% fetal bovine serum (FBS). The cells were seeded into 35-mm diameter dishes or 90-mm diameter dishes in 10% FBS and incubated at 37 °C in a humidified atmosphere of 5% CO_2_/95% air for 48 h.

### siRNA transfection

In order to knockdown HSP22 in MC3T3-E1 cells, the cells were transfected with HSP22-siRNA (#1 and #2) or negative control-siRNA (Neg) utilizing siLentFect Lipid Reagent (Bio-Rad Laboratories, Inc., Hercules, CA, USA) according to the manufacturer’s protocol. The cells were incubated with 50 nM siRNA (Neg, #1 or #2) siLentFect complexes for 24 h at 37 °C. The medium was then exchanged to α-MEM containing 0.3% FBS before experiments.

### Assay for IL-6 or VEGF

The siRNA-transfected MC3T3-E1 cells were stimulated by 10 μM of PGF2α or vehicle in 1 ml of α-MEM containing 0.3% FBS. The conditioned medium was collected after 48 h, and the concentration of IL-6 or VEGF was measured using the mouse ELISA kit for IL-6 or VEGF, respectively, according to the manufacturer’s protocol. The levels of IL-6 or VEGF were adjusted for the total protein levels of whole-cell lysates.

### Western blotting

The MC3T3-E1 cells transfected with siRNA were stimulated by 10 μM of PGF2α or vehicle in 1 ml of α-MEM containing 0.3% FBS for the indicated periods. The cells were then washed twice with phosphate-buffered saline, and then lysed, homogenized, and sonicated in a lysis buffer containing 62.5 mM Tris/HCl, pH 6.8, 2% sodium dodecyl sulfate (SDS), 50 mM dithiothreitol, and 10% glycerol. SDS-polyacrylamide gel electrophoresis (PAGE) was performed by the method of Laemmli [26] in 10% polyacrylamide gels. The protein was fractionated and transferred onto an Immun-Blot polyvinyl difluoride sheet (Bio-Rad, Hercules, CA, USA). The sheets were blocked with 5% fat-free dry milk in Tris-buffered saline-Tween (TBS-T; 20 mM Tris-HCl, pH 7.6, 137 mM NaCl, 0.1% Tween 20) for 1 h before incubation with primary antibodies. Western blot analysis was performed as described previously [27] using phospho-specific p44/p42 MAP kinase antibodies, p44/p42 MAP kinase antibodies, phospho-specific p38 MAP kinase antibodies, p38 MAP kinase antibodies, or GAPDH antibodies as primary antibodies with peroxidase-labeled antibodies raised in goat against rabbit IgG used as secondary antibodies. The primary and secondary antibodies were diluted at optimal concentrations with 5% fat-free dry milk in TBS-T. The peroxidase activity on the sheet was visualized on X-ray film using an ECL Western blotting detection system as described in the manufacturer’s protocol. The densitometric analysis was performed using a scanner and image analysis software package (image J version 1.48; NIH, Bethesda, MD, USA). The background-subtracted signal intensity of each phosphorylation signal was normalized to the GAPDH signal.

### Determination

Concentrations of protein in soluble extracts were estimated with a protein assay kit (Thermo Scientific, Rockford, IL, USA).

### Statistical analysis

The data were analyzed by analysis of variance followed by the Bonferroni method for multiple comparisons between pairs. The values of *p* < 0.05 were considered to be statistically significant. Each data are presented as the mean ± S.E.M. of triplicate determinations from three independent cell preparations.

## Results

### Effect of PGF2α-on the IL-6 release in HSP22-knockdown MC3T3-E1 cells

**Fig. 1:**
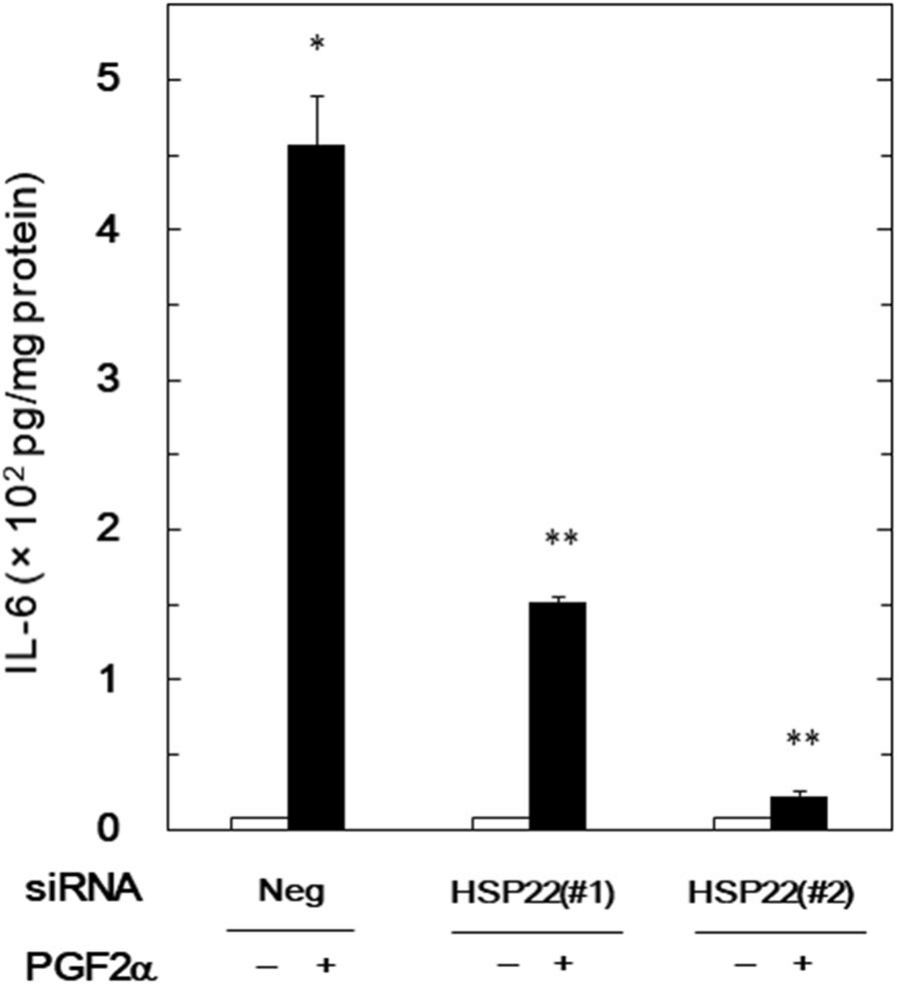
Effect of HSP22-knockdown on the PGF2α-induced release of IL-6 in MC3T3-E1 cells. The cells were transfected with 50 nM of HSP22-siRNA (#1 or #2) or negative control-siRNA (Neg) for 24 h and then stimulated by 10 μM of PGF2α or vehicle for another 48 h. IL-6 concentrations in the conditioned medium were determined. The values were presented as the levels adjusted for the total protein levels of whole-cell lysates. The data were analyzed by analysis of variance followed by Bonferroni method for multiple comparisons between pairs. The values of *p* < 0.05 were considered to be statistically significant. Each value represents the mean ± S.E.M. of triplicate determinations from three independent cell preparations. *p < 0.05 compared to the value of negative control-siRNA-transfected cells without PGF2α-stimulation. **p < 0.05 compared to the value of the negative control-siRNA-transfected cells with PGF2α-stimulation

In our previous study, we have shown that PGF2α induces the IL-6 synthesis in osteoblast-like MC3T3-E1 cells [7]. In order to investigate whether or not HSP22 is implicated in the IL-6 synthesis by PGF2α in these cells, we examined the effect of HSP22-knockdown on the PGF2α-elicited IL-6 release. We have already found that HSP22 was truly expressed in quiescent MC3T3-E1 cells, and the protein levels were markedly suppressed by the transfection of HSP22-siRNA (#1 and #2) [23]. The PGF2α-elicited IL-6 release in the HSP22-siRNA (#1 and #2)-transfected MC3T3-E1 cells was significantly reduced compared with that in the control cells (Fig. 1).

### Effect of PGF2α-on the VEGF release in HSP22-knockdown MC3T3-E1 cells

**Fig. 2:**
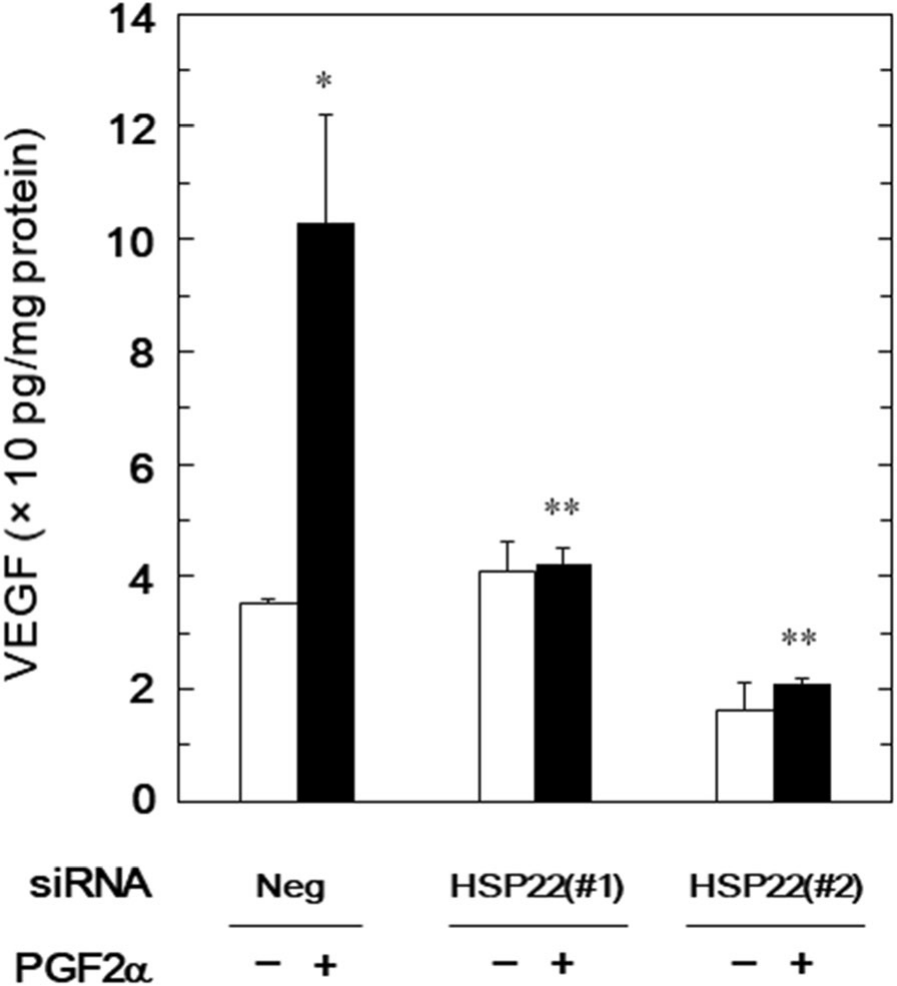
Effect of HSP22-knockdown on the PGF2α-induced release of VEGF in MC3T3-E1 cells. The cells were transfected with 50 nM of HSP22-siRNA (#1 or #2) or negative control-siRNA (Neg) for 24 h and then stimulated by 10 μM of PGF2α or vehicle for another 48 h. VEGF concentrations in the conditioned medium were determined. The values were presented as the levels adjusted for the total protein levels of whole-cell lysates. The data were analyzed by analysis of variance followed by Bonferroni method for multiple comparisons between pairs. The values of *p* < 0.05 were considered to be statistically significant. Each value represents the mean ± S.E.M. of triplicate determinations from three independent cell preparations. *p < 0.05 compared to the value of negative control-siRNA-transfected cells without PGF2α-stimulation. **p < 0.05 compared to the value of the negative control-siRNA-transfected cells with PGF2α-stimulation

We have demonstrated that PGF2α stimulates the synthesis of VEGF in addition to IL-6 in osteoblast-like MC3T3-E1 cells [8]. To clarify the involvement of HSP22 in the PGF2α-elicited VEGF synthesis, we examined the effect of PGF2α on the VEGF release in the HSP22-knockdown MC3T3-E1 cells. The levels of PGF2α-elicited VEGF release from the cells transfected with HSP22-siRNA (#1 and #2) were significantly lower than that from the control cells (Fig. 2).

### PGF2α-stimulated phosphorylation of p44/p42 MAP kinase or p38 MAP kinase in HSP22-knockdown MC3T3-E1 cells

**Fig. 3:**
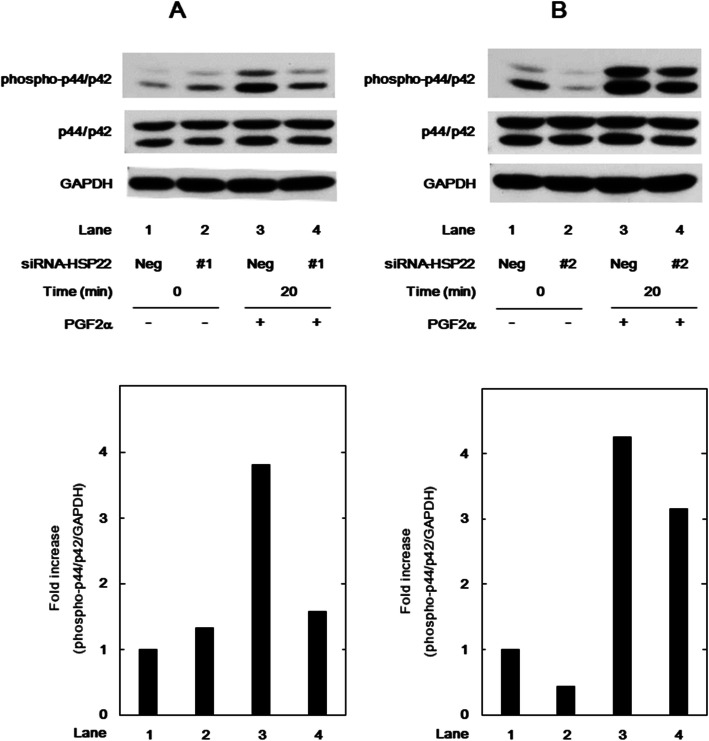
Effect of HSP22-siRNA on the PGF2α-stimulated phosphorylation of p44/p42 MAP kinase in MC3T3-E1 cells. The cells were transfected with 50 nM of HSP22-siRNA (#1) (**a**), HSP22-siRNA (#2) (**b**), or negative control-siRNA (Neg) for 24 h and then stimulated by 10 μM of PGF2α or vehicle for 20 min. The cell lysates were analyzed by Western blot analysis with antibodies against phosphor-specific p44/p42 MAP kinase, p44/p42 MAP kinase, or GAPDH. The histogram shows the quantification data of the phosphorylated levels after normalization with GAPDH levels determined by laser densitometry analysis. The level was plotted as the fold increase in comparison with that of the control cells without PGF2α-stimulation

We have previously shown that p44/p42 MAP kinase positively regulate the PGF2α-elicited synthesis of IL-6 and VEGF in osteoblast-like MC3T3-E1 cells [8, 10]. Therefore, to investigate whether or not HSP22 affects the PGF2α-induced activation of p44/p42 MAP kinase in these cells, we examined the effect of HSP22 knockdown on the phosphorylation of p44/p42 MAP kinase by PGF2α. The PGF2α-induced levels of phosphorylated p44/p42 MAP kinase in the HSP22-siRNA (#1 and #2)-transfected cells were markedly decreased compared with those in the control cells (Fig. 3a and b).

**Fig. 4:**
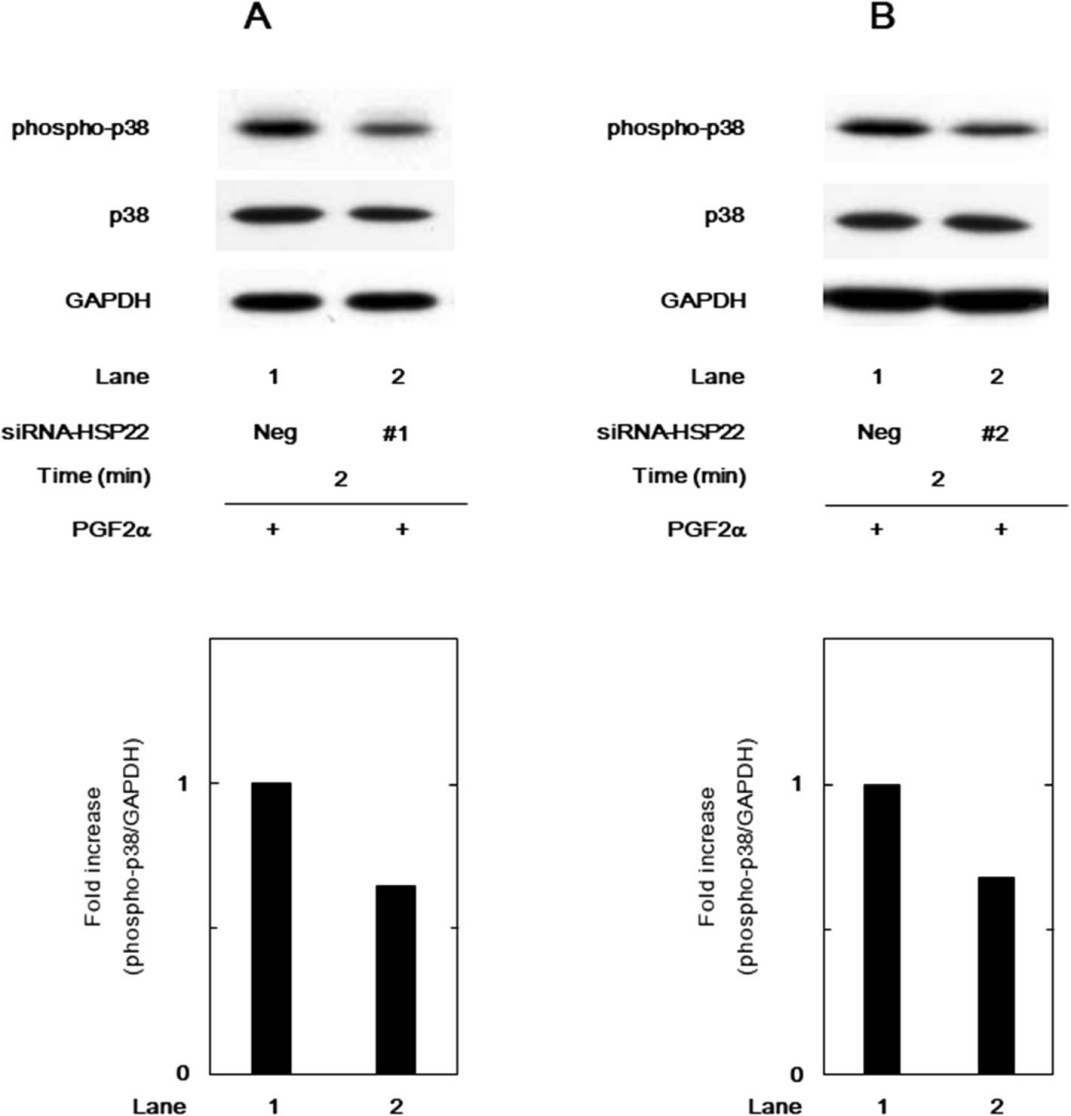
Effect of HSP22-siRNA on the PGF2α-stimulated phosphorylation of p38 MAP kinase in MC3T3-E1 cells. The cells were transfected with 50 nM of HSP22-siRNA (#1) (**a**), HSP22-siRNA (#2) (**b**), or negative control-siRNA (Neg) for 24 h and then stimulated by 10 μM of PGF2α for 2 min. The cell lysates were analyzed by Western blot analysis with antibodies against phosphor-specific p38 MAP kinase, p38 MAP kinase, or GAPDH. The histogram shows the quantification data of the phosphorylated levels after normalization with GAPDH levels determined by laser densitometry analysis. The level was plotted as the value of lane 1 to be 1

With regard to the intracellular signaling of PGF2α in osteoblasts, we have reported that p38 MAP kinase in addition to p44/p42 MAP kinase plays as a positive regulator in the synthesis of IL-6 and VEGF in MC3T3-E1 cells [11, 12]. We further examined the effect of PGF2α on the phosphorylation of p38 MAP kinase in the HSP22-knockdown cells. The HSP22-siRNA (#1 and #2) transfection remarkably reduced the PGF2α-stimulated levels of phosphorylated p38 MAP kinase compared with the control cells (Fig. 4a and b).

## Discussion

**Fig. 5:**
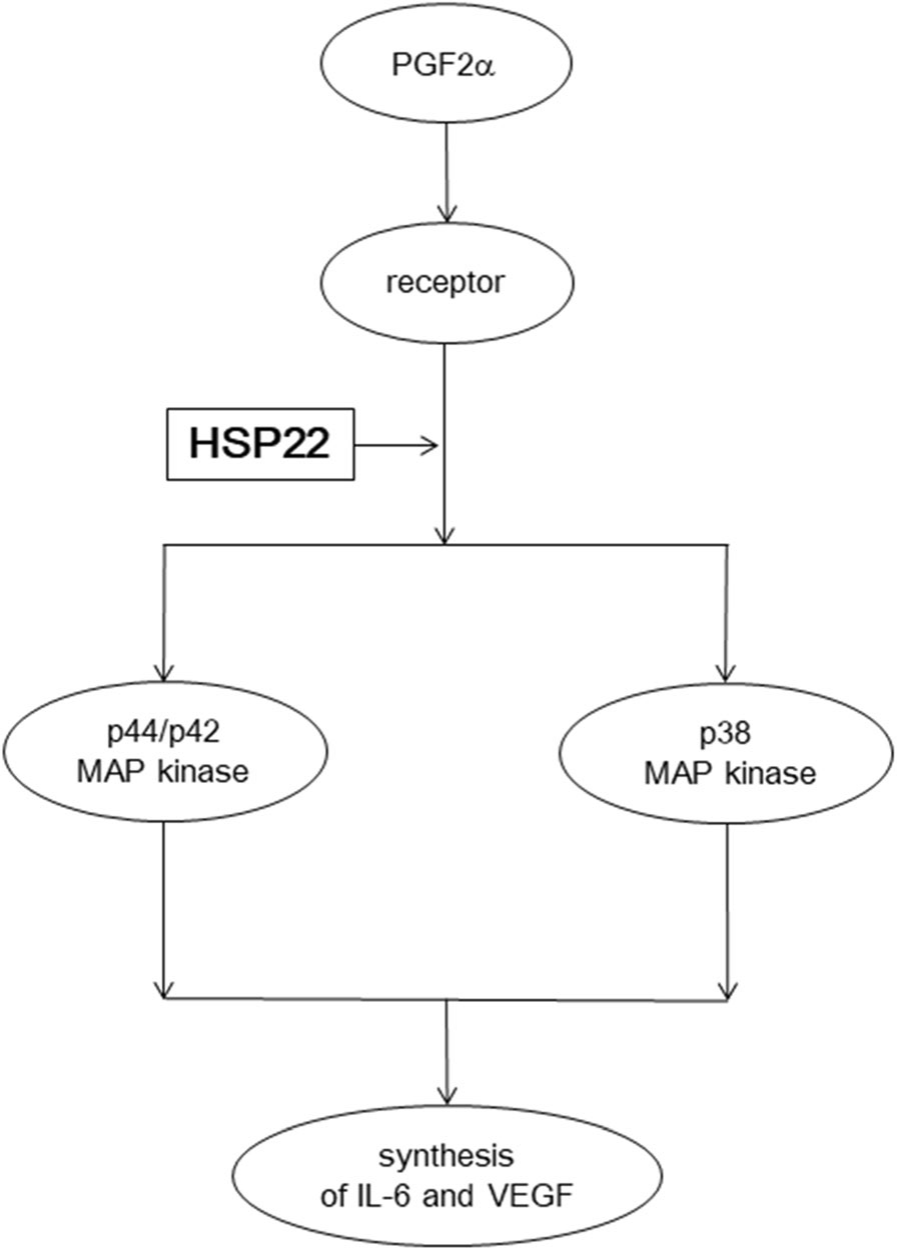
Schematic illustration of the role of HSP22 in the PGF2α-induced synthesis of IL-6 and VEGF in osteoblasts. HSP22, heat shock protein 22; PGF2α, prostaglandin F2α; MAP kinase, mitogen-activated protein kinase; IL-6, interleukin-6; VEGF, vascular endothelial growth factor

In the present study, we showed that the PGF2α-stimulated release of IL-6 and VEGF was reduced by HSP22-knockdown in osteoblast-like MC3T3-E1 cells. We have previously demonstrated that PGF2α induces the expression of IL-6 mRNA [28] and VEGF mRNA [8] in these cells. In addition, we have confirmed that the transfection of HSP22-siRNA downregulates the expression levels of HSP22 protein in MC3T3-E1 cells [23]. Based on these results, it seems likely that HSP22 positively regulates the PGF2α-induced synthesis of IL-6 and VEGF in MC3T3-E1 cells. As for the intracellular signaling of PGF2α in osteoblasts, we have reported that PGF2α stimulates the synthesis of IL-6 and VEGF through the activation of p44/p42 MAP kinase in osteoblast-like MC3T3-E1 cells [8, 10]. We showed herein that the level of phosphorylated p44/p42 MAP kinase induced by PGF2α was markedly attenuated in the HSP22-knockdown MC3T3-E1 cells. Thus, it seems likely that HSP22 is involved in the activation of PGF2α-induced p44/p42 MAP kinase in MC3T3-E1 cells. Additionally, we have reported that p38 MAP kinase acts as a positive regulator not only in the IL-6 synthesis by PGF2α but also in the VEGF synthesis in MC3T3-E1 cells [11, 12]. The HSP22 knockdown remarkably downregulated the PGF2α-stimulated level of phosphorylated p38 MAP kinase. Thus, these results suggest that HSP22 positively regulates the PGF2α-induced activation of p44/4p2 MAP kinase and p38 MAP kinase. Taking our findings into account as a whole, it is most likely that HSP22 functions at a point upstream of p44/p42 MAP kinase and p38 MAP kinase in osteoblast-like MC3T3-E1 cells, resulting in the regulation of PGF2α-stimulated synthesis of IL-6 and VEGF. The possible mechanism of HSP22 in PGF2α-induced synthesis of IL-6 and VEGF in osteoblasts shown here is summarized as Fig. 5.

In bone metabolism, IL-6 has been considered as a potent bone resorptive agent, which stimulates osteoclastic bone resorption [4]. On the other hand, accumulating current evidence indicates that IL-6 acts as an osteotropic factor under the condition of increased bone turnover in addition to the effect on bone resorption [29]. It is generally recognized that proinflammatory cytokines including IL-6 play a pivotal role in the pathogenesis of bone metabolic diseases including osteoporosis [30]. In the patients with rheumatoid arthritis, IL-6 causes chronic inflammation and bone loss [4]. It has been reported that HSP22 is abundantly expressed in synovial tissue in rheumatoid arthritis [31]. We have found that HSP22 exists in the unstimulated osteoblast-like MC3T3-E1 cells [23]. In the present study, we showed that HSP22 plays as a positive regulator in the PGF2α-induced synthesis of IL-6. Furthermore, we recently reported that tumor necrosis factor-α-stimulated synthesis of IL-6 is suppressed by HSP22 knockdown in MC3T3-E1 cells [24]. Taking these findings into account, it is most likely that HSP22 affects the IL-6 production in osteoblast and the development of rheumatoid arthritis. Thus, HSP22 inactivation in osteoblasts could suppress the loss of bone mass through the reduction of IL-6 synthesis. On the other hand, VEGF is a potent angiogenic factor which induces proliferation of endothelial cells [5]. It is currently recognized that the microvasculature provided by capillary endothelial cells is indispensable for bone remodeling [32]. In the present study, we demonstrated that HSP22 could positively regulate the PGF2α-stimulated synthesis of VEGF in osteoblasts. Based on these findings, it seems that HSP22 activity in osteoblasts might modulate the microvasculature through VEGF, resulting in the regulation of bone metabolism. Further experiments in vivo using animal are required to confirm the exact roles of HSP22 in bone metabolism. It has been reported that HSP22 knockout mouse was generated and used for the study of heart failure, showing that HSP22 deletion in HSP22 knockout mouse does not affect cardiac structure and function under normal conditions but precipitates heart failure under pressure overload [33]. However, to the best of our knowledge, there are no reports showing the exact roles of HSP22 in bone metabolism using the knockout mouse. Therefore, further studies to elucidate the role of HSP22 on the synthesis of IL-6 and VEGF which can lead to bone resorption or bone formation in animal experiments are necessary. Taken together, our results strongly suggest that HSP22 acts as a positive regulator in the PGF2α-induced synthesis of IL-6 and VEGF through p44/p42 MAP kinase and p38 MAP kinase in osteoblasts.

## Conclusion

In conclusion, we are the first to demonstrate that HSP22 (HSPB8) positively regulates PGF2α-induced synthesis of IL-6 and VEGF through p44/p42 MAP kinase and p38 MAP kinase pathways in osteoblasts. Our study showing that HSP22 could function as a stimulator of the PGF2α-stimulated IL-6 and VEGF synthesis in osteoblasts probably provides a novel insight with regard to HSP22 as an essential regulator of bone remodeling.

## Data Availability

All data generated or analyzed during this study are included in this published article.

## Abbreviations

HSP22: Heat shock protein 22
PGF2α: Prostaglandin F2α
IL-6: Interleukin-6
VEGF: Vascular endothelial growth factor
MAP: Mitogen-activated protein
GAPDH: Glyceraldehyde-3-phosphate dehydrogenase
α-MEM: α-Minimum-essential medium
FBS: Fetal bovine serum
SDS: Sodium dodecyl sulfate
TBS-T: Tris-buffered saline-Tween
